# Continental drift and plateau uplift control origination and evolution of Asian and Australian monsoons

**DOI:** 10.1038/srep40344

**Published:** 2017-01-13

**Authors:** Xiaodong Liu, Buwen Dong, Zhi-Yong Yin, Robin S. Smith, Qingchun Guo

**Affiliations:** 1 SKLLQG, Institute of Earth Environment, Chinese Academy of Sciences, Xi’an, 710075, China; 2 CAS Center for Excellence in Tibetan Plateau Earth Sciences, Beijing, 100101, China; 3 National Centre for Atmospheric Science, University of Reading, Reading, RG6 6BB, UK; 4 Department of Environmental & Ocean Sciences, University of San Diego, San Diego, 92110, USA

## Abstract

Evolutions of Asian and Australian monsoons have important significance for understanding the past global change but are still a controversial subject. Here, we explore systematically the effects of plate movement and plateau uplift on the formation and evolution of the Asian and Australian monsoons by numerical simulations based on land-sea distributions and topographic conditions for five typical geological periods during the Cenozoic. Our results suggest that the timings and causes of formation of the monsoons in South Asia, East Asia and northern Australia are different. The Indian Subcontinent, which was located in the tropical Southern Hemisphere in the Paleocene, was influenced by the austral monsoon system simulated at that time. Once it moved to the tropical Northern Hemisphere in the Eocene, the South Asian monsoon established and remained persistently thereafter. However, the monsoons of East Asia and northern Australia did not appear until the Miocene. The establishment of the simulated low-latitude South Asian (northern Australian) monsoon appeared to have strongly depended on the location of mainland India (Australia), associated with northward plate motion, without much relation to the plateau uplift. On the contrary, the establishment of the mid-latitude East Asian monsoon was mainly controlled by the uplift of Tibetan plateau.

Asian and Australian monsoons are a prominent component of atmospheric circulation and exert a significant influence on the global climate^1^,^2^,^3^. The onset and development of the Asian and Australian monsoons have played important roles in the formation and evolution of regional climates and environments during the Cenozoic^3^,^4^,^5^,^6^,^7^,^8^,^9^,^10^,^11^,^12^. However, the origin of monsoons is still a controversial subject in the scientific community^4^,^5^,^6^,^7^,^8^,^9^,^10^,^11^,^12^,^13^. There is still conflicting evidence regarding the timings of establishment of the monsoon climates in South Asia^4^,^5^,^6^, East Asia^7^,^8^,^9^,^10^,^11^, and northern Australia^12^,^13^. For example, the change in vegetation from forests to grassland in northern Pakistan^4^ has been considered as important evidence of the formation of the South Asian monsoon during the late Miocene (7–8 Ma), while recent proxies of monsoon circulation from the western Arabian Sea suggested that the monsoon first appeared in the mid-Miocene (~12 Ma) and was fully established in the late Miocene^5^. Moreover, lacustrine deposits under a coal mine in Rajasthan of northwestern India indicated the presence of a monsoon-type climate in the early Eocene (55–52 Ma)^6^. In East Asia, loess-soil sequences in the Chinese loess deposits, representing alternating episodes of dominant winter and summer monsoons, were initially interpreted as suggesting that the East Asian monsoon first started in the early Quaternary (~2.5 Ma)^7^. This age was extended back to the late Miocene (~8 Ma) based on the eolian red clay deposits beneath the loess^8^. Later, with the discovery of the early Miocene loess^9^, comparisons with the Cenozoic vegetation/environmental changes in China^10^ and comprehensive reconstruction of Asian paleoenvironmental patterns^11^, the formation of the East Asian monsoon was constrained to the time around the end of the Oligocene to the beginning of the Miocene (~22 Ma). In contrast, geological evidence indicates the presence of the northern Australian monsoon in the Southern Hemisphere (SH) in the late Quaternary^12^,^13^.

During the Cenozoic, plate movements in the Asian-Australian region have been very active^14^, characterized by the northward motion of the Indian Subcontinent and Australia^15^,^16^ and corresponding tectonic activity, especially the uplift of the Tibetan Plateau (TP)^17^,^18^,^19^,^20^. This activity made the TP and its surroundings into a region of the intensive interactions between the different components of the earth system^21^. Therefore, for a long time, the formation of the Asian monsoons has been attributed to the uplift of the TP^22^,^23^ as well as changes in the land-sea distribution^24^,^25^. However, at this time, different opinions exist regarding the origin of monsoon climates, especially the Asian monsoons, and the timings of their establishment, partially stemming from a large amount of numerical simulation results. For example, some numerical experiments emphasized the impact of the TP uplift as it is likely to have enhanced the Asian summer and winter monsoons in the late Cenozoic^22^, prompted enhancements of the East and South Asian monsoons in the late Miocene^8^, or caused the formation of the monsoon climate in the northern part of East Asia^23^. Furthermore, it has been suggested that the phased and sub-regional uplift of the TP^17^, especially the northern TP, had different effects on regional climates and environments^26^, and may be more closely related to the origin and evolution of the monsoon climate in the northern part of East Asia^26^. Other simulation studies have pointed out that the changes in land-sea distribution may have had equally important effects on the evolution of the Asian monsoons. For example, the retreat of the Paratethys may have caused the enhancement of the Asian monsoons in the early Oligocene^24^ and the establishment of early-Miocene monsoon-based paleo-environmental patterns in China^25^. A recent simulation study^27^ argued that the Asian monsoons may have occurred in the late Eocene when high atmospheric CO_2_ levels effectively intensified the global hydrological cycle and increased monsoon precipitation, and thereby compensated for the effects of a lower TP at that time.

Even though the Asian-Australian region is recognized as where the most prominent changes in land-sea distribution occurred during the Cenozoic^14^,^15^,^16^, the effects of plate movement on the origins of the Asian-Australian monsoons have not been examined in a systematic way. More importantly, while many past numerical experiments examined the effects of the TP uplift on the formation and evolution of the Asian monsoons^8^,^22^,^23^,^26^, most of them ignored the changes in land-sea configuration caused by plate motion. In other words, the combined effects of plateau uplift and the plate movements have not been fully evaluated.

Given the geological facts that the significant movement of the Indian-Australian plate^14^,^15^,^16^ and the large-scale uplift of the Tibetan Plateau (TP)^17^,^18^,^19^,^20^ occurred during the Cenozoic, we designed a series of numerical experiments using a coupled atmospheric-oceanic general circulation model (AOGCM)^28^ to systematically examine the origins and evolution of the Asian and Australian monsoons as well as the relative roles and combined effects of plate motion and topography in 5 typical Cenozoic geological periods: mid-Paleocene (MP: ~60 Ma), late-Eocene (LE: ~40 Ma), late-Oligocene (LO: ~25 Ma), late-Miocene (LM: ~10 Ma), and present day (PD: 0 Ma) (See Methods and Supplementary Information for details of the model and design of experiments).

## Results

**Figure 1:**
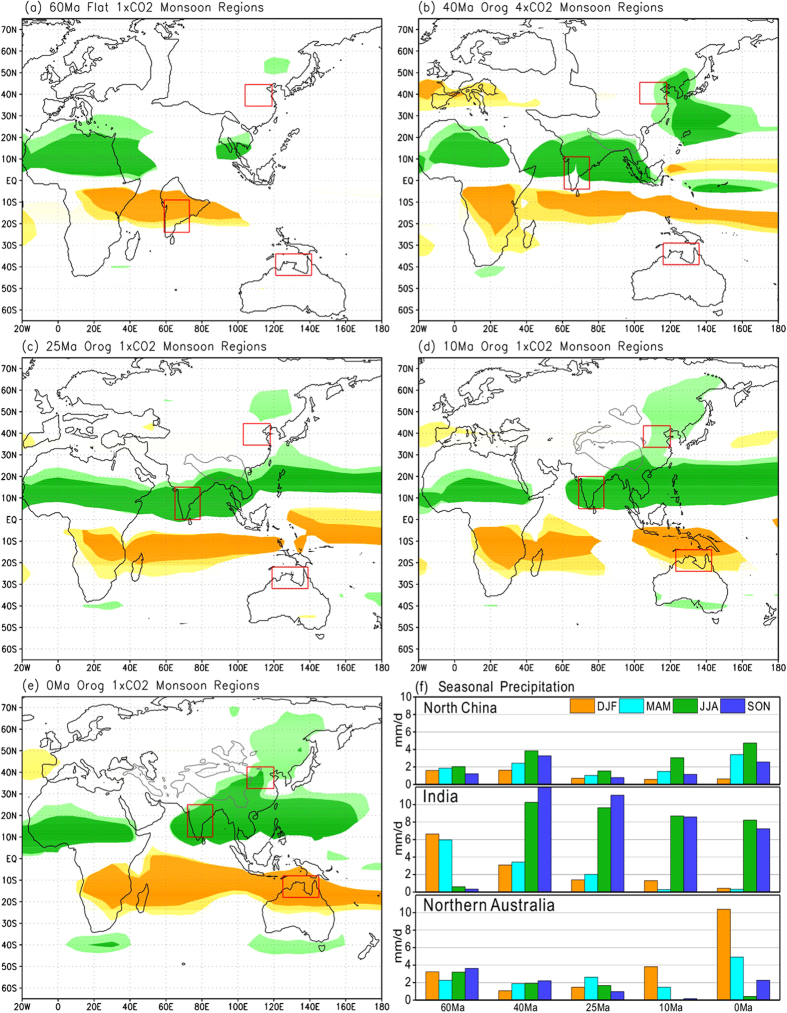
Simulated monsoon regions over eastern hemisphere and seasonal mean precipitation over North China, India, and northern Australia during the 5 geological periods. (**a**) mid-Paleocene (MP: ~60 Ma), (**b**) late-Eocene (LE: ~40 Ma), (**c**) late-Oligocene (LO: ~25 Ma), (**d**) late-Miocene (LM: ~10 Ma), and (**e**) present day (PD: ~0 Ma). The light-dark green (yellow-orange) areas indicate monsoon regions with the rainy season in JJA (DJF), in which the darker shades represent the typical monsoon regions. In the light (dark) green areas, the rainfall difference between boreal summer (JJA) and boreal winter (DJF) is greater than 200 (400) mm and JJA rainfall is more than 40% of the annual total. In the yellow (orange) areas, the rainfall difference between austral summer (DJF) and austral winter (JJA) is greater than 200 (400) mm and DJF rainfall is more than 40% of the annual total. Red boxes indicate North China, India, and northern Australia where seasonal (DJF, MAM, JJA and SON) mean precipitations are shown in (**f** ). Note that the geographic coordinates of these regions were changing over time, but their relative positions corresponding to the specific land masses remained the same. Maps were created using GrADS (Version 1.9b4, http://cola.gmu.edu/grads/).

**Figure 2:**
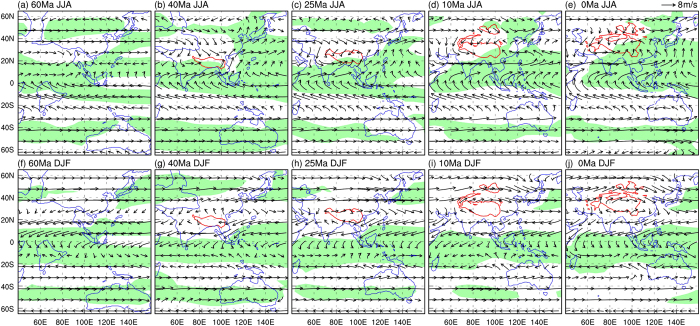
Simulated 850 wind vector (m s^−1^) and precipitation (mm) fields in JJA (top) and DJF (bottom) during the 5 geological periods. (**a**) MP in JJA, (**b**) LE in JJA, (**c**) LO in JJA, (**d**) LM in JJA, (**e**) PD in JJA, (**f**) MP in DJF, (**g**) LE in DJF, (**h**) LO in DJF, (**i**) LM in DJF, (**j**) PD in DJF. Regions where seasonal precipitation greater than 300 mm are shaded. Blue lines are coast lines and red lines outline regions where orography is higher than 1500 m. Maps were created using GrADS (Version 1.9b4, http://cola.gmu.edu/grads/).

Since the beginning of the Cenozoic, the Asian-Australian monsoon region has seen great changes. In MP, the Indian Subcontinent was dominated by the simulated Southern Hemisphere (SH) monsoon system (Fig. 1a) since it was positioned in the tropical SH. In June-July-August (JJA), the entire Indian Subcontinent was south of the Intertropical Convergence Zone (ITCZ) and dominated by the southeasterly trade winds with relatively low rainfall (Figs 1f and 2a). In December-January-February (DJF), a segment of the ITCZ shifted southward to central India, so that northern India was dominated by the northwesterly winds as the Northern Hemisphere (NH) northeasterly trade winds crossed the equator into the SH, while central India experienced its rainy season (Figs 1f and 2f). This suggests that when India was located in the tropical SH, the monsoon climate there was mainly due to the seasonal shift of the ITCZ (Figs 1a and 2a,f) with the wet season in DJF and March-April-May (MAM) and the dry season in JJA and September-October-November (SON) (Fig. 1f). Mid-latitude East Asia and Australia were under the influence of the westerlies year-round without any monsoon presence (Figs 1a and 2a,f) while the typical monsoon climate only existed around the Indochina Peninsula.

By LE, the Indian Subcontinent moved into the NH and was connected to the Eurasian continent and the TP was under the early stage of uplift (Figs S1b and S2b – the prefix “S” indicates content from the Supplementary Information). The simulated typical monsoon region expanded to approximately 20°N in South Asia, covering northern and central India, and the Bay of Bengal (Fig. 1b). Southern India experienced a humid tropical climate due to its proximity to the equator and was influenced by the southwesterly winds which originated from the SH bringing rainfall in boreal summer (Fig. 2b), while northern India was relatively dry in winter due to the northeasterly winds which originated from higher latitudes (Fig. 2g). These circulation features and the strong seasonality of enhanced rainfall in JJA and SON over India (Fig. 1f and Table S8) suggest that the South Asian monsoon could have established as early as LE. Since the Asian land mass significantly expanded in size due to the Indian Subcontinent joining Eurasia, a strong low pressure system with cyclonic circulation pattern developed over Mongolia in summer caused by intense land surface heating (Fig. 2b). The associated southwesterly flows brought moisture into East Asia, creating humid conditions for the region centered on the Korean Peninsula (Figs 1b and 2b). It is worth noting that the mid-latitude southerly flows, which did not come directly from the low-latitude oceans, were mainly related to the enhanced atmospheric CO_2_ as they existed even if there was no topography (Fig. S6f) as discussed in the following. In contrast, this region was under the influence of westerlies in winter and had much drier conditions (Fig. 2g). Therefore, it could be classified as a monsoon region based only on precipitation seasonality (Fig. 1b). Meanwhile, Australia was still located south of 30°S without any monsoon climate (Fig. 1a,f).

In LO, as the Indian Subcontinent pushed further northward and the Tethys Sea reduced in size, the tropical land-sea configuration had changed significantly by this time (Figs S1 and S2). In the corresponding simulation the Indian Subcontinent was entirely covered by the typical monsoon climate with further enhanced rainfall seasonality (Fig. 1c,f and Table S8). The monsoon circulation over India and a westerly-dominated circulation over North China (Fig. 2c,h) did not change much from those in LE. Based on the fact that weak annual rainfall and weak seasonality were typical in North China (Fig. 1f and Table S8), we can infer that the mid-latitude East Asian monsoon was not yet established at this time. The rainy area near the Korean Peninsula in LE (Fig. 2b) disappeared in LO (Fig. 2c) due to reduced atmospheric CO_2_ and then moisture in the atmosphere. Although Australia continued to move northward, it was still dominated by the SH westerlies year-round and did not show the presence of monsoon (Figs 1c,f and 2c,h).

By LM, the Indian Subcontinent had only slightly shifted further to the north and the simulated monsoon climate remained essentially unchanged from LO (Fig. 1). The greatest change occurred in East Asia. With the continued uplift of the TP, especially its growth to the north^19^,^20^ (Fig. S2), the East Asian monsoon region extended from South to North China (Fig. 1d) with enhanced boreal summer and winter monsoon circulations (Fig. 2d,i) and rainfall seasonality (Fig. 1f and Table S8). Another major change occurred in Australia with its northernmost part now under the dominance of the SH monsoon system (Fig. 1d), characterized by southeasterly winds from the SH subtropics in JJA and northwesterly winds from the equator in DJF (Fig. 2d,i) with enhanced rainfall seasonality (Fig. 1f and Table S8). These results suggest that the complete establishment of the entire modern Asian-Australian monsoon system most likely happened after LM.

From LM to PD, India and the entire South Asian monsoon region saw little change, while the East Asian monsoon region expanded further northward (Figs 1e and 2e) due to the uplift of the northern TP, Pamir, Tienshan Mountains, and the Mongolian Plateau^19^,^20^,^26^. A typical monsoon climate in the NH is now found in North Africa, the Indian Subcontinent, Indochina, the southeastern TP, eastern China, and the tropical western Pacific. In the SH, the typical monsoon climate covers a continuous zone from tropical southern Africa to northern Australia, East Timor, and Papua New Guinea with further increased rainfall seasonality over northern Australia (Fig. 1f and Table S8). These modeling results in general match the observed patterns based on actual rainfall measurements^2^.

**Figure 3:**
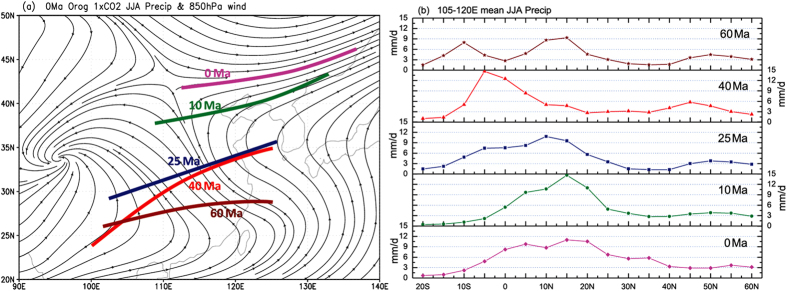
Northern boundary of the East Asian summer (JJA) monsoon circulation and meridional distribution of mean summer rainfall during the 5 geological periods. (**a**) Northern limits of the East Asian monsoon overlaid on the 850 hPa streamline field of PD. (**b**) summer rainfall rates (mm day^−1^) averaged over 105°–120°E. The corresponding ages are labeled for each period in both (**a**) and (**b**). Longitudes 105°–120°E cover the conventional range of the present-day East Asian monsoon region. Maps were created using GrADS (Version 1.9b4, http://cola.gmu.edu/grads/).

Because of the unique nature of the East Asian monsoon, we specifically examined the northward expansion of the boreal summer monsoon circulation and rain-belt in East Asia during the Cenozoic (Figs 3 and S4). As the northern boundary of the East Asian summer monsoon circulation progressed northward from the southern coast of China (~26°N) in MP to North China (~42°N) in PD (Figs 3a and S4a–e), which also revealed the timing of establishment of the East Asian summer monsoon over mainland East Asia in LM (Figs 3a and S4d), the monsoon rain-belt also moved northward (Fig. 3b). There were two rainfall peaks in the tropics (10°S and 15°N) in MP (Fig. 3b) corresponding to the double-ITCZ at the time, while the subtropical low-rainfall belt was found between 30–40°N. Since LE, the peak tropical rainfall latitude continued to migrate northward from 5°S to 10°N in LO and then to 15°N in LM and PD (Fig. 3b). From LO to LM, summer rainfall in the mid-latitudes (20–40°N) started to increase, but the most prominent increase in summer rainfall in the mid-latitude East Asia occurred from LM to PD (Fig. 3b) corresponding to the full establishment of the boreal summer monsoon circulation over mainland East Asia (Fig. S4d,e). The latitudinal gradient of rainfall rates may indicate the path of moisture transport. In LE, the 35–45°N rainfall gradient was directed from north to south, while the gradient in PD was from south to north (Fig. 3b). Therefore, relatively high rainfall in mid-latitude East Asia during LE was not caused by moisture transport originated from the tropical ocean.

**Figure 4:**
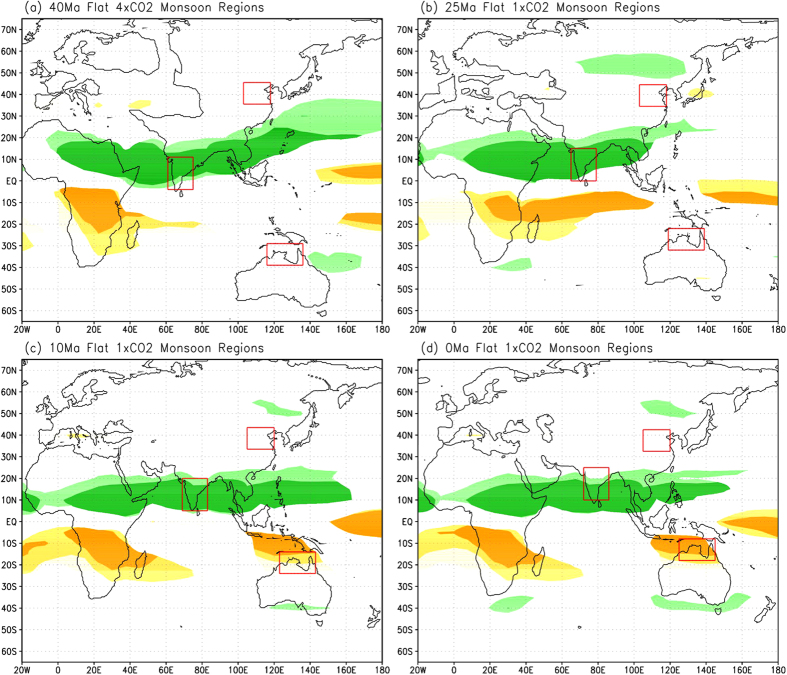
Simulated monsoon regions over eastern hemisphere for experiments without orography during the 4 geological periods. (**a**) LE, (**b**) LO, (**c**) LM, and (**d**) PD. The light-dark green (yellow-orange) areas indicate monsoon regions with the rainy season in JJA (DJF) where summer-winter rainfall difference is greater than 200 mm and summer rainfall is more than 40% of the annual total. Darker shades represent the typical monsoon regions where summer-winter rainfall difference is greater than 400 mm. Maps were created using GrADS (Version 1.9b4, http://cola.gmu.edu/grads/).

The above results suggest that the establishment of the South Asian and northern Australian monsoons was closely related to the northward movement of the Indian-Australian plate. In contrast, the land-sea distribution for East Asia remained relatively stable and, therefore, the establishment and evolution of the mid-latitude East Asian monsoon were much more dependent on the phased uplift of the TP. Comparisons between the experiments with and without global topography (Tables S6 and S7) reveal that during LE to PD, the South Asian monsoon would still exist without the influence of topography (Fig. 4); although under the PD condition, the monsoon in northern India would be located slightly to the south if the influence of topography were not present (Fig. 4d). Similarly, the monsoon region in northern Australia during LM and PD would also exist without the topography although its size was reduced somewhat (Fig. 4c,d) as compared to that with topography (Fig. 1d,e). In contrast, if there was no topography, westerly circulation would dominate almost the entire mainland East Asia in summer (Fig. S5a–d) and the rainfall distribution pattern would have incurred major changes, especially during LM and PD (Fig. S6c,d) and, as the result, the East Asian monsoon would disappear in all geological periods (Fig. 4).

It should be pointed out that the precipitation-based “monsoon” region centered over the Korean Peninsula during LE (Fig. 1b) does not necessarily indicate the establishment of the East Asian monsoon, because the typical boreal summer monsoon circulation barely reached the Yangtze River valley at this time (Figs 3a and S4b). Enhanced summer rainfall here can be caused by topography (Fig. S6a) or elevated CO_2_ levels with the presence of topography (Fig. S6e). However, the precipitation pattern would disappear if there was no topography even with the same elevated CO_2_ level (Figs 4a and S6f). Instead, there would be a broad belt of westerly circulation and minor increases in summer rainfall in mid-latitude Eurasia (Fig. S6f). The effects of topography were manifested by the presence of a mid-latitude trough centered at ~100°E downwind from the low TP (Fig. S6a), and the corresponding southwesterly winds, rather than the typical East Asian summer monsoon circulation (as in Figs S4e and S6d), would bring increased rainfall to this region (Fig. S6a,e). Therefore, it is the uplift of the TP that caused the northward expansion of the East Asian summer monsoon circulation and rainfall to reach the areas north of the Yangtze River in LM, and then further to North and Northeast China (Figs 3 and S4d,e).

## Discussion

Our results suggest that South Asian, East Asian and Australian monsoons formed at different times, a conclusion also supported by various geological evidence^6^,^10^,^11^,^12^,^13^, and by different causes. The establishment of the South Asian monsoon may have occurred in late Eocene when the Indian Subcontinent moved into the tropical NH, while the northern Australian monsoon did not fully establish until the late Miocene. Therefore, an important conclusion from this study is that the timing of when India (Australia) moved into the tropical latitudes in the NH (SH) controlled the establishment of the South Asian (northern Australian) monsoon. The changes in paleo-latitudinal positions caused by plate motion controlled the transitions from non-monsoon to monsoon climates in India and Australia. In other words, the paleo-latitudinal position of a region determined the paleoclimate of that region, which was often true even before the Cenozoic^29^,^30^. On the other hand, the establishment of the mid-latitude East Asian monsoon was strongly dependent on the TP uplift, especially the uplift of the northern TP^23^,^26^ in the late Miocene.

This study emphasizes the importance of tectonic boundary conditions and relative contributions of the plate motion and topography uplift for the formation and evolution of the Asian-Australian monsoons. Some properties of the modern monsoon variability could not be simply extended to the geological time periods. For example, the present-day Australian summer monsoon is likely linked with the East Asian winter monsoon that is in turn related to the effect of TP^31^. However, our simulations show that the origin of the Australian monsoon mainly depends on the position of the Australian mainland, rather than the cross-equatorial flows from East Asia. Given the fact that there are still large uncertainties in the reconstruction of boundary conditions in geological times, some conclusions of this paper may need be further tested and verified with more geological records.

Other influencing factors and their comprehensive effects with tectonic boundary conditions deserve in-depth research. The elevated CO_2_ levels during the late Eocene may have caused increased precipitation in the mid-latitudes, but our results suggest that there did not exist a typical summer monsoon circulation over mainland East Asia during this time. Although our simulations suggest the enhancement and expansion of the East Asian summer monsoon under the condition when only the plate movement and plateau uplift are considered from LO to LM, some geological records^11^,^32^,^33^ show that the intensity of the East Asian summer monsoon decreased on the whole in the Miocene. Thus, other forcing factors, such as atmospheric CO_2_ concentration^32^,^33^,^34^, should also be considered in order to fully understand the long-term evolution of the Asian monsoons.

## Methods

### Climate model

We use a coupled Atmosphere Ocean General Circulation Model (AOGCM) to simulate climatic conditions in 5 geological periods during the Cenozoic to investigate the responses of the Asian and Australian monsoons to the individual and combined effects of land-sea configuration and plateau uplift associated global plate movement. The model used in this study is the Fast Met. Office and UK universities Simulator (FAMOUS AOGCM)^28^, which is a low-resolution version of the HadCM3 AOGCM^35^,^36^ and structurally almost identical to HadCM3. FAMOUS has an atmospheric component with a horizontal resolution of 5° × 7.5° and 11 vertical levels. The ocean component has a horizontal resolution of 2.5° × 3.75°, with 20 vertical levels. The atmosphere and ocean components are coupled once every day. FAMOUS can produce climate and climate-change simulations that are reasonably similar to HadCM3 without flux adjustments but runs much faster. This characteristic is particularly useful for long runs of paleoclimate simulations. More details of the description of FAMOUS and the simulated climate are documented previously^28^,^36^. Although the model’s spatial resolution is relatively low, its simulation of both present-day and future climate are in line with state of the art models in many respects and has the capability to simulate modern atmospheric processes realistically across the Asia-Australia region. For example, compared with the NCEP Reanalysis and CMAP observed precipitation data, FAMOUS accurately portrays the characteristics of the modern Asian monsoons, including the regional details on the 850hPa wind field and precipitation distribution (Fig. S3). Therefore, we are confident that the monsoon phenomena at the regional scale as represented in our paleoclimatic numerical experiments provide a reliable foundation for our analysis of the origin and evolution of the Asian-Australian monsoons.

### Design of numerical experiments

We first designed 5 basic experiments (Table S6) to portray the circulation patterns during the Cenozoic: Present-day (PD), late Miocene (LM), late Oligocene (LO), late Eocene (LE), and mid-Paleocene (MP). These experiments are described in detail in the Supplementary Information. Reconstructions of the corresponding land-sea distribution patterns and paleo-topography/bathymetry of these 5 geologic periods are presented in Figs S1 and S2. To investigate the causes of the changes in the circulation patterns associated with the origins and evolution of the Asian-Australian monsoons, we designed a set of comparative experiments (Exps 1a, 2a, 3a and 4a) to represent scenarios without global topography for PD, LM, LO, and LE respectively, while Exp. 4b represents a low CO_2_ scenario of LE with global topography and Exp. 4c characterizes also a low CO_2_ scenario of LE but with no global topography (Table S7). In analyzing the simulation results, the differences between Exps 1–4 and Exps 1a–4a reveal the effects of topography on the origin and evolution of the Asian-Australian monsoons, with each pair characterized by its specific land-sea distribution pattern during a given geologic period. The difference between Exp. 4 and Exp. 4b (Exp. 4a and Exp. 4c) represents the effect of elevated CO_2_ concentration during LE with (without) the presence of topography. These experiments allow us to examine the combined effects of the changes in the land-sea configuration due to plate movement and the uplift of the TP. All experiments have been run for 1000 model years and the last 100 year mean results are used in the analysis.

### Monsoon clarification in model simulations

Since monsoon climates are commonly characterized by alternating rainy and dry seasons, we first use precipitation amounts and seasonality to determine monsoon region distributions^2^. NH (SH) monsoon regions are defined as areas where the precipitation difference between boreal (austral) summer JJA (DJF) and boreal (austral) winter DJF (JJA) is greater than 200 mm and JJA (DJF) precipitation is more than 40% of the annual total. Further, among these monsoon regions, where summer-winter precipitation differences greater than 400 mm can be considered as the typical monsoon regions (Fig. 1). Changes in rainfall seasonality in three present-day monsoon regions (North China, India, and northern Australia) during the Cenozoic are also quantified for the 5 geological periods (Fig. 1f, Table S8). According to the traditional definition of monsoon climate, monsoons have a seasonally reversing wind system^37^,^38^, characterized by wet summers and dry winters. Therefore, we further analyze the lower troposphere (850 hPa) winter and summer circulation patterns to understand the evolution of the Asian-Australian monsoon system during the 5 simulated geological periods (Fig. 2). We also examine the northward expansion of the boreal summer monsoon circulation in East Asia as the TP uplift and growth proceeded during the Cenozoic (Fig. 3). On the 850 hPa surface, the northernmost position that can be reached by the warm and humid flows originated from the tropical ocean marks the northern limit of the East Asian summer monsoon circulation (Fig. S4), while the effects of topography on the summer circulation patterns in East Asia are presented in Fig. S5. In addition, we used the differences in summer rainfall between the comparative and basic experiments to analyze the effects of topography on the Asian-Australian monsoons during the Cenozoic, as well as the effects of elevated CO_2_ levels during LE (Fig. S6). The focus of this study is the combined effects of plate movement and TP uplift as the primary factors on the origins and evolution of the Asian-Australian monsoons, while the effects of other factors on the evolution of the monsoons, such as the changing atmospheric CO_2_ concentration, global ice volume, land surface and oceanographic processes, although not considered here, should be addressed in depth in future studies.

## Additional Information

**How to cite this article**: Liu, X. *et al*. Continental drift and plateau uplift control origination and evolution of Asian and Australian monsoons. *Sci. Rep.* **7**, 40344; doi: 10.1038/srep40344 (2017).

**Publisher's note:** Springer Nature remains neutral with regard to jurisdictional claims in published maps and institutional affiliations.

## Supplementary Material

Supplementary Information
