# Supplementary material for: Continental drift and plateau uplift control origination and evolution of Asian and Australian monsoons
Source: Sci Rep. 2017 Jan 13;7:40344. doi: 10.1038/srep40344 (PMC5233948; doi:10.1038/srep40344)
Supplement: Supplementary Information [file srep40344-s1.pdf]

Supplementary Information

Continental drift and plateau uplift control origination and evolution of Asian and Australian monsoons

Xiaodong Liu[1,2,*], Buwen Dong[3], Zhi-Yong Yin[4], Robin S. Smith[3] and Qingchun Guo[1]

[1] SKLLQG, Institute of Earth Environment, Chinese Academy of Sciences, Xi'an, 710075, China.

[2] CAS Center for Excellence in Tibetan Plateau Earth Sciences, Beijing, 100101, China.

[3] National Centre for Atmospheric Science, University of Reading, Reading, RG6 6BB, UK.

[4] Department of Environmental & Ocean Sciences, University of San Diego, San Diego, 92110, USA.

[*] e-mail: liuxd@loess.llqg.ac.cn

Reconstruction of Cenozoic land-sea distribution patterns. Plate reconstructions for this study were performed using GPlate, an open-source geological information model[39,40] developed by an international team with members from the US, Australia, and Norway (http://www.gplates.org/contact.html). This modeling tool contains a database of the positions and orientations of the modern land masses during different geological periods for the past 140Ma (Global EarthByte GPlates Coastlines)[39]. It has been used in a variety of studies, ranging from a global plate reconstruction for the past 200Ma[41], to more localized study on Antarctic circumpolar current 30Ma ago[42], as well as a tool for interactive 3-D visualization of global geological and geophysical data[43]. In our study, the reconstructions of the land-sea configurations were a two-step process. First we used the GPlate software to reposition and rotate the land masses and ocean basins to the positions corresponding to the 4 geological periods: mid-Paleocene (MP, ~60Ma), late Eocene (LE, ~40Ma), late Oligocene (LO, ~25Ma) and late Miocene (LM, ~10Ma) using the published rotation poles[44]. Then we edited the land mass boundaries using ArcGIS 10.0 (ESRI, Redlands) according to various sources[45-48]. For example, for the MP period, we reduced the area of the Indian Subcontinent[45]. For the LE and LO periods, we modified the collision boundary between the Indian Subcontinent and Eurasia[46]. We also modified the Mediterranean Tethys and Paratethys boundaries[47] for the LO period. For the LM period, we removed the Tethys Sea between the Arabian Peninsula and Eurasia[48]. The finally reconstructed global land-sea distribution for the 5 geological periods are presented in Fig. S1.

Reconstruction of Cenozoic TP and global paleo-topography/bathymetry. For global paleo-topographic and paleo-bathymetric reconstructions of the LM, we rotated and repositioned the modern topography and bathymetry (ETOPO2, http://www.ngdc.noaa.gov/ mgg/global/etopo2.html) and an existing reconstruction for 15Ma[49] to 10Ma using GPlates, and then estimated the paleo-topography by assuming a constant rate of elevation and bathymetric change. In other words, with a constant rate of change between 0Ma elevation (H0) and 15Ma elevation (H15), the paleo-elevation for 10Ma (H10) was estimated as H10 = H15 + (H0 - H15)*5/15. The reconstruction for the LE (~40Ma) was directly based on the rotation and reposition of the reconstruction for 45Ma (H45)[50]. Then the paleo-topography

of 25Ma was estimated in a similar way as H25 = H45 + (H15 - H45)*20/30. Finally, we modified major topographic and bathymetric features according to a large amount of geological evidence and overlaid the resulted paleo-topography/bathymetry on the corresponding reconstructed land-sea distributions using ArcGIS. For example, we modified the paleo-topography of the Rocky Mountains by first calculating the paleo-to-modern elevation ratios of different geological periods obtained from geological evidence (Table S1) and then applied the mean ratios of a given geological period to the modern topography to get the paleo-topography. The same approach was employed to estimate paleo-topography for the Andes Mountains (Table S2), Greenland and Antarctica (Table S3), and the Pamirs, Tienshan Mountains, Iran Plateau, and Mongolian Plateau (Table S4). For the paleo-topography of the Tibetan Plateau (TP), we first divided the TP into 5 terranes (Table S4). For each terrane block, we calculated the paleo-to-modern elevation ratios for each geological period and then applied the ratios to the modern TP topography to get the paleo-topography. The paleo-elevation data used in the reconstructions of the TP paleo-topography are listed in Table S5. Another consideration is the paleo-latitudinal positions of the TP and the Pamirs as determined by paleomagnetism and other geological evidence. For example, paleomagnetism evidence indicated that Dingri County of Tibet (29°N, 87°E at the present) was located at 11°N, 17°N, and 23°N during 40Ma, 25Ma, and 10Ma[51], respectively, while its longitudinal position did not change much over time. Other evidence suggested that the southern rim of the TP was located at 10-15°N during the Eocene[52]. Accordingly, we adjusted the latitudinal positions of the TP by moving it as a whole from south to north to its locations during LE, LO, and LM. Similarly, we modified the paleo-latitudes of the Pamirs, also representing a south-to-north movement over time[53]. The final reconstructed topographies and ocean bathymetries of the 5 geological periods are presented in Fig. S2.

Boundary conditions of the basic and comparative experiments. The two sets of experiments designed for this study were based on the standard pre-industrial setup of FAMOUS[28]. There were 5 basic experiments in the first set (Table S6): Exps. 1-5 corresponding to the 5 geological periods during the Cenozoic: Present-day (PD), late

Miocene (LM), late Oligocene (LO), late Eocene (LE) and mid-Paleocene (MP), using the reconstructed land-sea configurations and topography/bathymetry at 0Ma, ~10Ma, ~25Ma, ~40Ma, and ~60Ma, respectively, regridded onto the FAMOUS grids and smoothed appropriately. To simplify the physical processes and allow us to focus on the effects of land-sea configuration and topography, we used the pre-industrial CO_2 level of 280 ppmv for all but the LE period (Exp. 4), during which the CO_2 level was set as 4×pre-industrial level in reference to existing geological records[54] and previous simulation work[27]. Taking into account the fact that large-scale tectonic uplifts of TP mainly occurred in the Eocene or later (Table S5), the terrains in the MP experiment were set to 0 elevation for the sake of simplicity. In addition, all vegetation and soil characteristics for all experiments were set to globally uniform values everywhere except Antarctica, each characteristic at each gridpoint being given the global average for non-glaciated surfaces in the pre-industrial simulation of FAMOUS.

The second set of numeric simulations included 6 comparative experiments (Table S7). Exps. 1a–4a used the same land-sea configurations as in Exps. 1–4, but the global topography was set to zero or the sea level. Additionally, there were two extra experiments 4b and 4c for the LE period. The only difference between Exp. 4b and Exp. 4 was the CO_2 level, which was set as 280 ppmv in Exp. 4b as compared to 4×280 ppmv in Exp. 4, while Exp. 4c presents a low CO_2 condition (280 ppmv) without global topography. All experiments with topography are abbreviated as “OROG”, while all zero-topography experiments as “FLAT” in the following.

Changes in the East Asian summer monsoon circulation and the causes. An important consideration when defining the East Asian monsoon is the alternating seasonal winds originated from the tropical ocean and interior of the land mass. The climate model used in this study showed good performance in simulating distributions of the East Asian regional circulation and precipitation (Fig. S3). Since the northernmost limit of the summer 850 hPa winds originating from the tropical ocean marks the northern boundary of the typical summer monsoon circulation, it can be used to illustrate the northward expansion of the monsoon region in East Asia during the Cenozoic. Figure S4 shows the progressively

northward migration of the northern boundary of the summer monsoon circulation from the southern coast of China (~26°N) in MP (~60Ma), to roughly the Yangtze River valley (~32°N) in LE (~40Ma) and LO (~25Ma), and then further to North China in LM (~10Ma) and finally reaching north of 42°N in PD (0Ma). Prior to 25 Ma (Figs S4a-c), the majority of the East Asian land mass, especially North China, was dominated by the westerly circulation in summer. During LE (40 Ma), there was a strong low pressure system centered at ~110°E, 55°N and the southwesterly winds in the southern part of this system produced increased summer rainfall over the Korean Peninsula and Japan Islands (Fig. 1b). However, this circulation pattern does not qualify as the typical summer monsoon circulation for East Asia as the monsoon winds originating from the tropical ocean did not reach the region beyond the Yangtze River valley at this time (Fig. S4b). In other words, a simple increase in summer precipitation does not necessarily indicate the presence of a typical monsoon climate. Only after 10 Ma, meridional (southerly) winds progressed northward beyond 35°N (Fig. S4d) and eventually reached their modern position of ~42°N (Fig. S4e).

Forcings of the changes in summer rainfall in East Asia. Topography played an important role in shaping the modern East Asian monsoon circulation. Figure S5 shows the results of the experiments for the same land-sea configuration as in Fig. S4, but with no global topography. In these cases, there was barely any area over the mainland East Asian that could be defined as being controlled by a typical summer monsoon circulation. This means that for East Asia, as the land-sea configuration remained relatively stable during the Cenozoic, the uplift of the TP (and global topography) was critical for the establishment of the East Asian monsoon. Without the topography, summer monsoon circulation would not have existed even under the present-day land-sea configuration (Fig. S5d). Changes in the summer circulation pattern caused by removal of topography are reflected in distributions of rainfall in East Asia (Figs S6a-d), as the differences in summer rainfall between the basic experiments (Exp. 1-4, Table S6) and the corresponding comparative experiments (Exp. 1a-4a, Table S7) during LE, LO, LM and PD. Without topography in LE (Fig. S6a), summer rainfall over northern India and Bay of Bengal would decrease significantly while Korea and Japan would also see reductions. The impact of topography was greatest over the Bay

of Bengal in LO (Fig. S6b) while mainland East Asia did not see much change in summer rainfall at all. The greatest changes were observed for LM and PD if the topography were removed (Figs S6c,d), corresponding to the time of establishment and further enhancement of the East Asian monsoon. In the meantime, Fig. S6e (i.e., Exp. 4 minus Exp. 4b) suggests that the effects of high CO_2 concentration during LE are mostly manifested in the enhanced summer rainfall in northern India, Bay of Bengal, and over the tropical oceans south of 15°N, with only limited impact on the summer rainfall over mainland East Asia. Fig. S6f (i.e., Exp. 4a minus Exp. 4c), however, indicates that without topography, the area of high summer rainfall centered at the Korean Peninsula would disappear and be replaced by a broad region of increased rainfall in mid-latitude East Asia, most likely corresponding to the elevated atmospheric humidity during this period.

Table S1: Paleo-elevations of the Rocky Mountains

Time (Ma)	Location	Paleo-elevation (m)	Present elevation (m)	Percentage (%)	References
40	Wyoming	3000	2500	120	[55]
35	Wyoming	2500	2500	100	[56]
40	Cordillera	3000-4000	2500	120-160	[57]
65	Cordillera	2400-4800	2500	96-192	[57]
65	Cordillera	4000	2500	160	[58]

Table S2: Paleo-elevations of the Andes Mountains

Time (Ma)	Location	Paleo-elevation (m)	Present elevation (m)	Percentage (%)	References
10	Puna Plateau	3300	3600	91	[59]
14	Eastern Cordillera	2500	4000	62	[59]
10.7	Central Andes	1850	3700	50	[60]
20	Central Andes	1220	3700	33	[60]
19−25	Chucal	1000	4200	24	[61]
13.8−20.8	Potosi	0-1320	4300	0-31	[62]
15	Atacama Desert	2750	4500	44-67	[63]
7.6	Bolivian Altiplano	1400	3800	18-55	[64]
11.5−10.3	Bolivian Altiplano	1700	3800	45	[64]
6.8	Bolivian Altiplano	3850	3800	76-103	[64]
6−7	Pislepampa	1300	3600	33-39	[65]
15	Sal. de Tequendama	0-500	2475	0-20	[66]

Table S3: Paleo-elevations of Greenland and Antarctica

Region	Time	References
Greenland	25Ma	[45]
Greenland	10Ma	[45]
Antarctica	25Ma	[45, 67- 69]
Antarctica	10Ma	[69]

Table S4: Percentages of paleo-to-modern elevations of the 5 terranes of the TP for different geological periods

Time (Ma)	Location	Percentage (%)
40	Qiangtang block	70
40	Lhasa block	70
40	Songpan-Ganze and Kunlun block	40
40	Himalaya block	1
40	Qaidam and Qilian block	34
25	Qiangtang block	75
25	Lhasa block	75
25	Songpan-Ganze and Kunlun block	59
25	Himalaya block	33
25	Qaidam and Qilian block	53
10	Qiangtang block	83
10	Lhasa block	83
10	Songpan-Ganze and Kunlun block	83
10	Himalaya block	88
10	Qaidam and Qilian block	83

Table S5: Paleo-elevation data used in reconstruction of paleo-topography of the TP

Serial number	Location	Time (Ma)	Paleo-elevation (m)	References
1	Kunlun Pass	40	2040	[70]
2	Hoh Xil	39	4000	[18]
3	Lunpola Basin	40	4800	[71]
4	Lunpola Basin	40	1000	[71]
5	Nima Basin	40	2000	[71]
6	Zhada Basin	40	0	[71]
7	Thakkhola Graben	40	500	[71]
8	Gyirong Basin	40	1000	[71]
9	Tetang Fm.	40	500	[71]
10	Thakkhola Graben	40	4800	[71]
11	Tetang Fm.	51-28	4923	[72]
12	Gyirong Basin	38	2400	[73]
13	Namling-Oiyug Basin	38	4000	[73]
14	Namling-Oiyug Basin	40	4400	[74]
15	Namling-Oiyug Basin	40	800	[75]
16	Lhasa	40	400	[76]
17	Hoh Xil Basin	40	1600	[76]
18	Kunlun	40	1200	[76]
19	Himalaya	40	3295-3494	[77]
20	Qaidam	40	2000	[78]
21	Qilian	40	3500	[79]
22	Jiuquan	50	3000-4000	[80]
23	Qiangtang terranes	45-35	4800	[81]
24	Lhasa	45-35	1000	[81]
25	Hoh Xil Basin	45-35	2000	[81]
26	Himalaya	45-35	500	[81]
27	Qaidam	45-35	1000	[81]
28	Qilian	45-35	0	[81]
29	Jiuquan	45-35	4800	[81]
30	Qiangtang terranes	51-48	4000	[82]
31	Yepokangra	40	3300	[39]
32	Ali (Zanda)	40-37	5000	[83]
33	Woma	26	4700	[84]
34	Paiku Co	25	4638	[85]
35	Jiagula	20	4800	[71]
36	Puli (Yadong graben)	20	4000	[71]
37	Yali (north of Nyalam)	20	0	[71]
38	Heihuling	20	2000	[71]
39	Heihuling	20	3000	[71]
40	Hoh-Xil	20	500	[71]

Table S6: Boundary conditions in five basic experiments

	Exp.1	Exp.2	Exp.3	Exp.4	Exp.5
Geologic time	PD	LM	LO	LE	MP
Land-sea	0 Ma	10 Ma	25 Ma	40 Ma	60 Ma
Orography	0 Ma	10 Ma	25 Ma	40 Ma	FLAT
CO_2	280ppm	280ppm	280ppm	4×280ppm	280ppm

Table S7: Boundary conditions in six comparative experiments

	Exp.1a	Exp.2a	Exp.3a	Exp.4a	Exp.4b	Exp.4c
Geologic time	PD	LM	LO	LE	LE	LE
Land-sea	0 Ma	10 Ma	25 Ma	40 Ma	40 Ma	40 Ma
Orography	FLAT	FLAT	FLAT	FLAT	40 Ma	FLAT
CO_2	280ppm	280ppm	280ppm	4×280ppm	280ppm	280ppm

Table S8: Percentages of seasonal mean precipitation to annual totals (%) in three areas (India, North China, and northern Australia) for the five geological times

Times	Areas	DJF	MAM	JJA	SON
MP ~60Ma	N. China	23.6	28.1	30.5	18
	India	48.3	44.5	4.5	2.4
	N. Australia	25.9	18.6	26.1	29.3
LE ~40Ma	N. China	14.3	21.9	34.8	29.2
	India	10.6	12	36	41.5
	N. Australia	14.8	27.1	27.3	31
LO ~25Ma	N. China	17.1	25.8	38.3	19.1
	India	5.7	8.5	40.2	45.8
	N. Austra	21.5	39.4	25.1	14.2
LM ~10Ma	N. China	8.8	24	49.2	18.4
	India	6.8	1.6	46.5	45.4
	N. Australia	68.6	27	0.6	3
PD ~0Ma	N. China	5.5	30.4	42	22.6
	India	2.7	1.9	51.1	44.5
	N. Australia	57	27.5	2.3	12.6

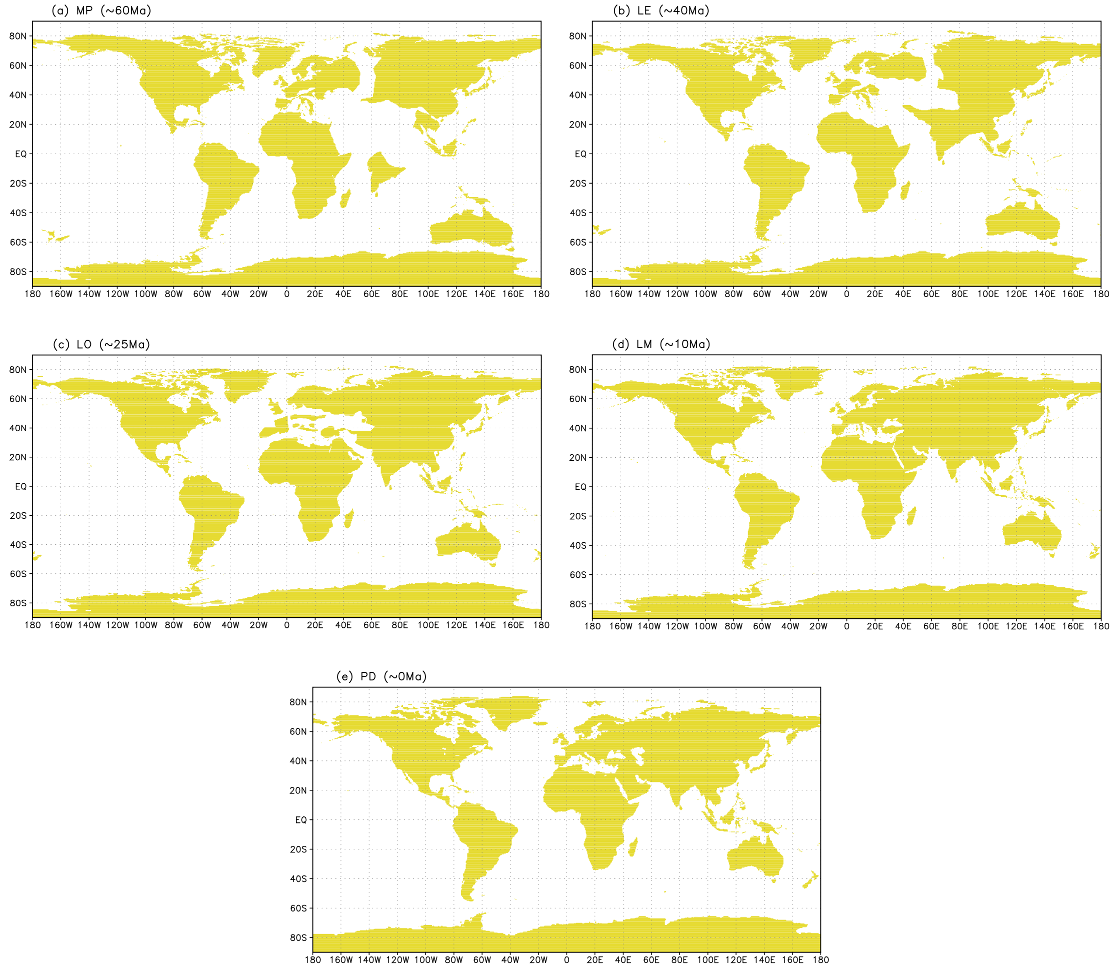

Figure S1: Reconstructed land-sea distributions of the 5 geological periods. (a) for mid-Paleocene (MP), (b) for late-Eocene (LE) , (c) for late-Oligocene (LO), (d) for late-Miocene (LM), and (e) for present day (PD). Maps were created using GrADS (Version 1.9b4, http://cola.gmu.edu/grads/).

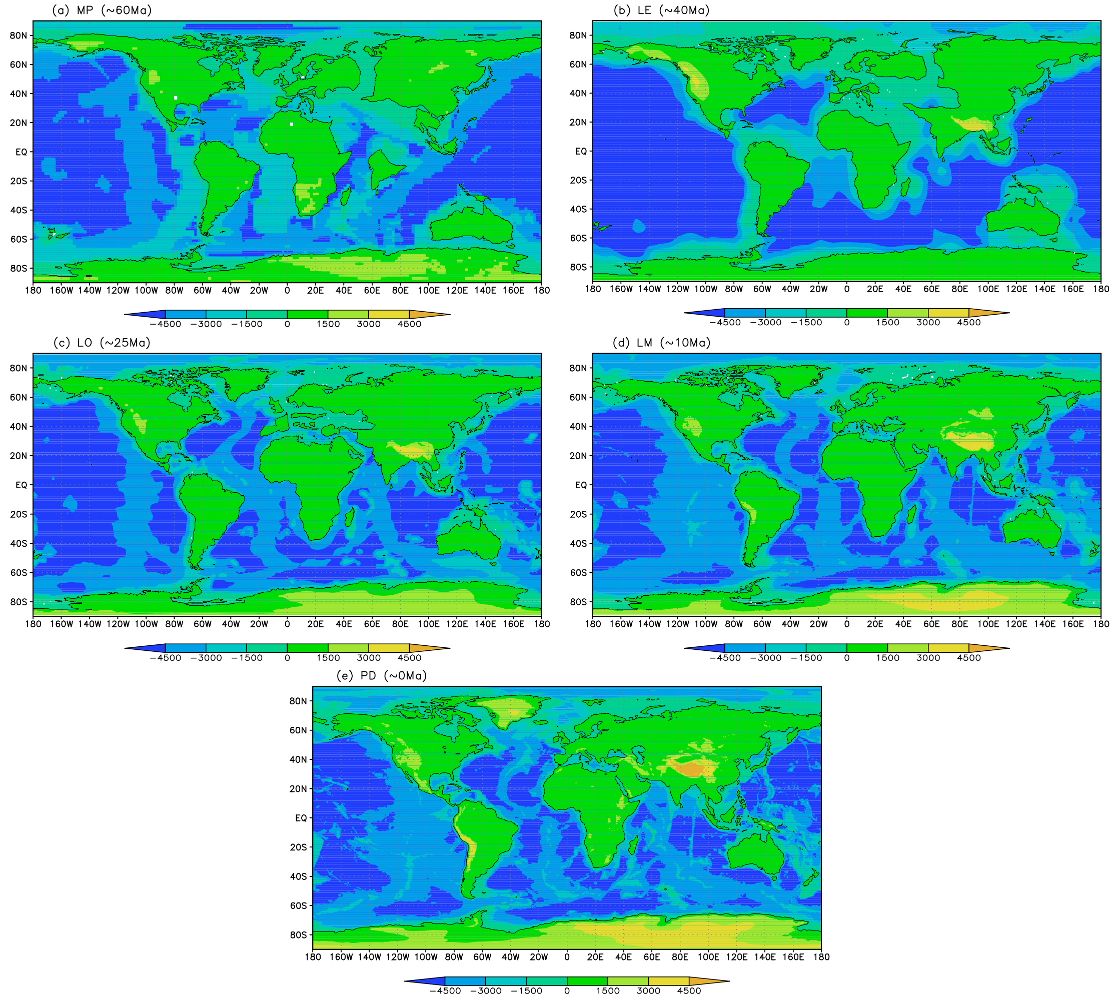

Figure S2: Reconstructed topographies and ocean bathymetries (meters) of the 5 geological periods. (a) MP, (b) LE, (c) LO, (d) LM, and (e) PD. Maps were created using GrADS (Version 1.9b4, http://cola.gmu.edu/grads/).

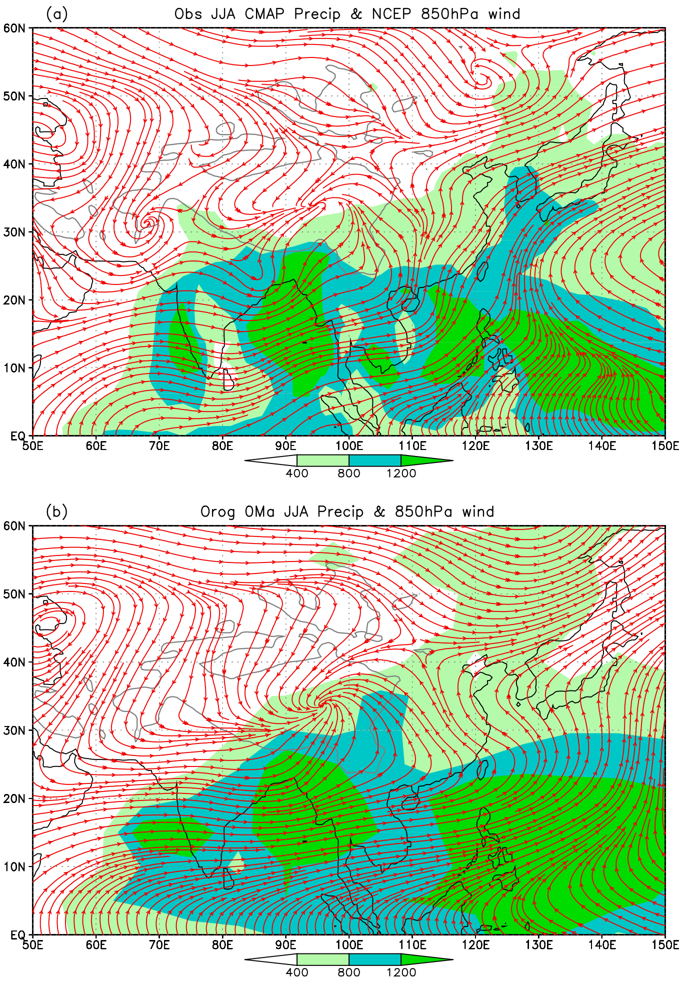

Figure S3: The spatial patterns of JJA climatology for observations and model simulation. (a) 850 hPa streamline fields from NCEP reanalysis and precipitation (shaded, mm) from CMAP. (b) model simulation for present-day JJA. Maps were created using GrADS (Version 1.9b4, http://cola.gmu.edu/grads/).

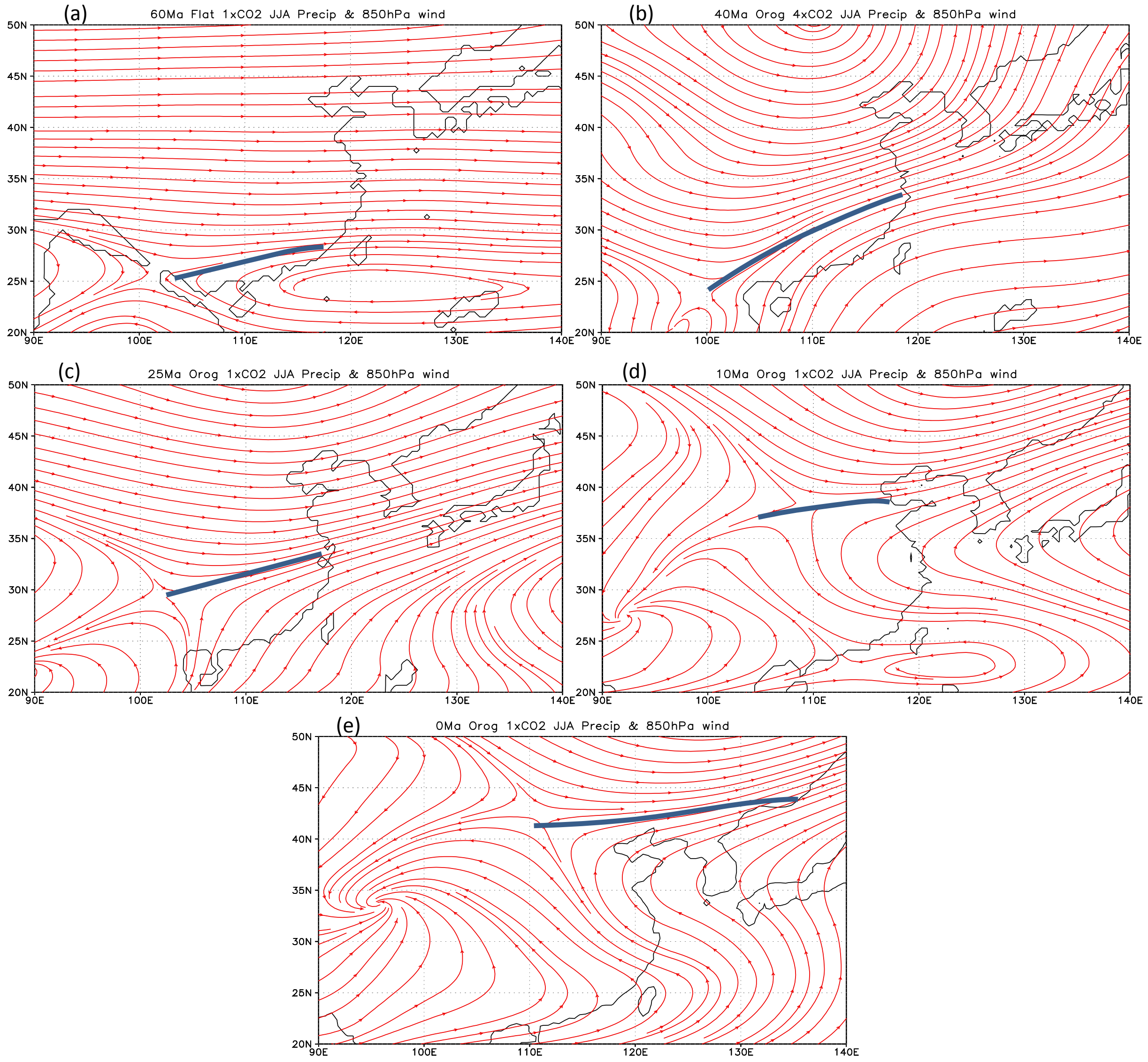

Figure S4: Time evolution of the streamline fields (red lines) at 850 hPa and northern boundary (thick blue lines) of East Asian summer monsoon during the 5 geological periods (Table S6). On the 850 hPa surface, the northernmost position that can be reached by the warm and humid flows originated from the tropical ocean marks the northern limit of the East Asian summer monsoon circulation. (a) for MP, (b) for LE, (c) for LO, (d) for LM, and (e) for PD. The northern limit of the summer monsoon is defined as the areas that could be reached by the warm and humid flows originated from the tropical ocean. Maps were created using GrADS (Version 1.9b4, http://cola.gmu.edu/grads/).

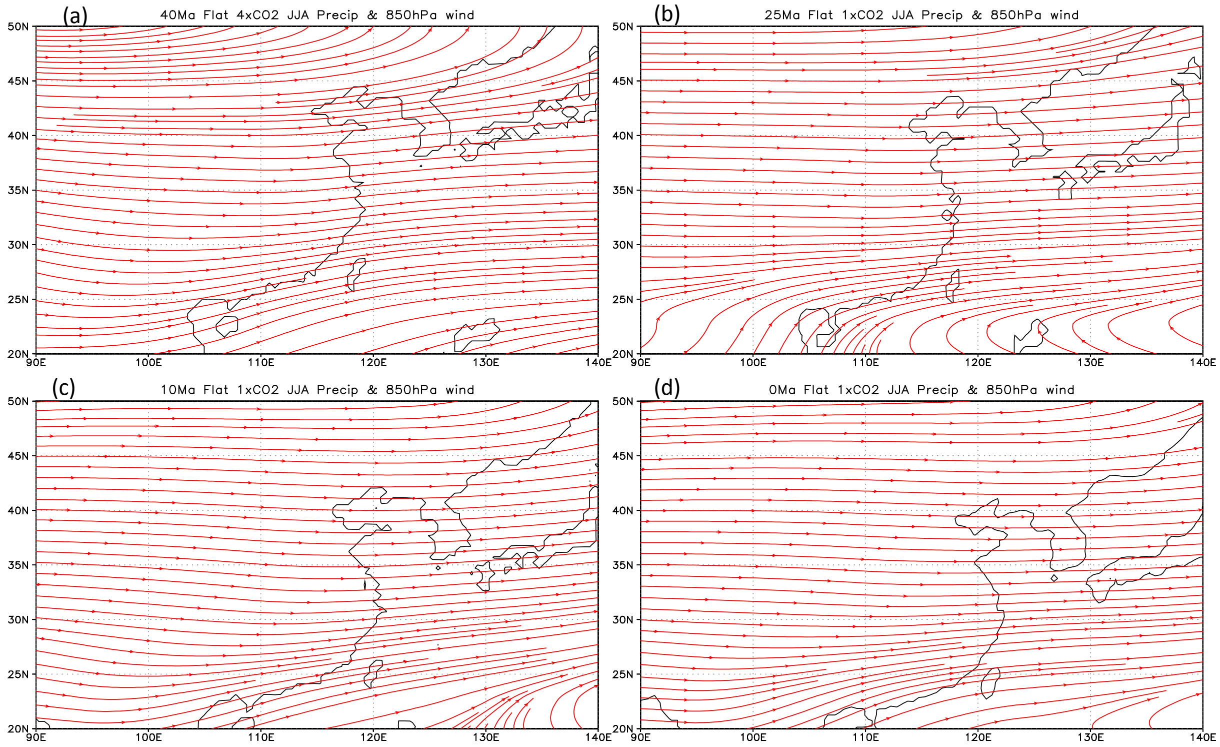

Figure S5: Time evolution of the streamline (red lines) at 850 hPa in experiments with flat orography during the 4 geological periods (Table S7). (a) for LE, (b) for LO, (c) for LM, and (d) for PD. There was barely any area over the East Asian land mass that can be defined as being dominated by a true summer monsoon circulation. Maps were created using GrADS (Version 1.9b4, http://cola.gmu.edu/grads/).

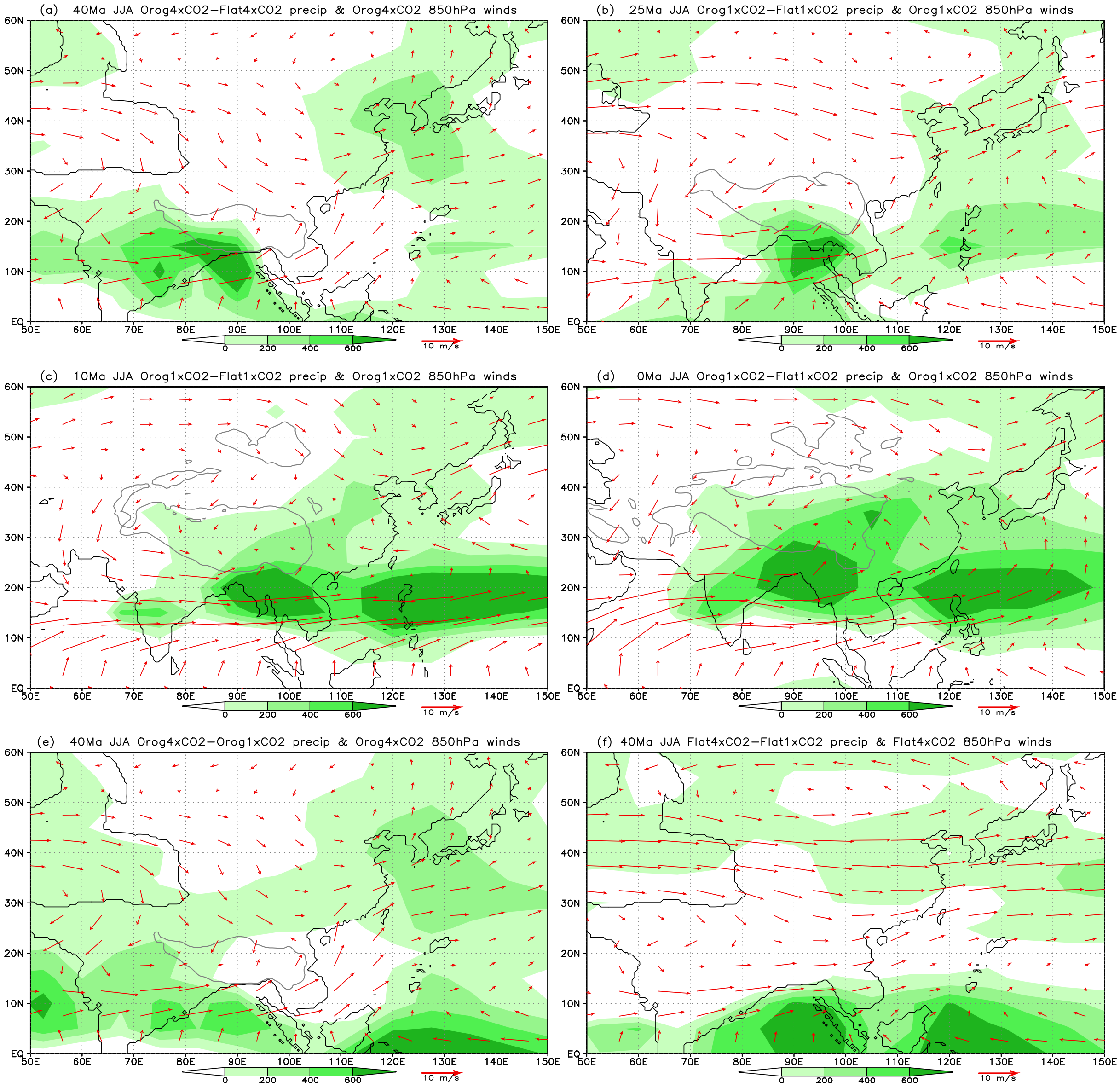

Figure S6: Changes in summer precipitation over Asia and their causes. Illustrated are the 850 wind field (m s^{-1}) and in precipitation changes (mm) resulted from increased topography or atmospheric CO_2 concentration. (a)-(d): 850 hPa wind fields for four geological periods (LE, LO, LM, and PD) and precipitation changes resulted from topography. (e) 850 hPa wind during LE and precipitation changes resulted from 4×CO_2 with topography. (f) 850 hPa wind during LE without topography and precipitation changes resulted from 4×CO_2 under condition of no topography. Maps were created using GrADS (Version 1.9b4, http://cola.gmu.edu/grads/).

Supplementary References

39. Gurnis, M. *et al*. Plate tectonic reconstructions with continuously closing plates. *Comput. Geosci*. **38**, 35-42 (2012).
40. Qin, X. *et al*. The GPlates geological information model and markup language. *Geosci. Instrum. Method. Data Syst.* **1,** 111-134 (2012).
41. Seton, M. *et al*. Global continental and ocean basin reconstructions since 200 Ma. *Earth Sci. Rev*. **113**, 212-270 (2012).
42. Scher, H. D. *et al*. Onset of Antarctic Circumpolar Current 30 million years ago as Tasmanian Gateway aligned with westerlies. *Nature* **523,** 580-583 (2015).
43. Müller, R. D., Flament, N., Matthews, K. J., Williams, S. E. & Gurnis, M. Formation of Australian continental margin highlands driven by plate–mantle interaction. *Earth Planet. Sci. Lett.* **441**, 60-70 (2016).
44. Müller, R. D., Sdrolias, M., Gaina, C. & Roest, W. R. Age, spreading rates, and spreading asymmetry of the world's ocean crust. *Geochem. Geophys. Geosyst.* **9,** Q04006, doi:10.1029/2007GC001743 (2008).
45. Zachos, J., Pagani, M., Sloan, L., Thomas, E. & Billups, K. Trends, rhythms, and aberrations in global climate 65 Ma to present. *Science* **292,** 686-693 (2001).
46. Sun, Z. *et al*. Palaeomagnetism of Late Cretaceous sediments from southern Tibet: Evidence for the consistent palaeolatitudes of the southern margin of Eurasia prior to the collision with India. *Gondwana Res.* **21,** 53-63 (2012).
47. Popov, S. V. *et al*. Late Miocene to Pliocene palaeogeography of the Paratethys and its relation to the Mediterranean. *Palaeogeogr. Palaeoclimatol. Palaeoecol.* **238,** 91-106 (2006).
48. McQuarrie, N., Stock, J. M., Verdel, C. & Wernicke, B. P. Cenozoic evolution of Neotethys and implications for the causes of plate motions. *Geophys. Res. Lett.* **30**, 2036, doi:10.1029/2003GL017992 (2003).
49. Herold, N., Seton, M., Müller, R. D., You, Y. & Huber, M. Middle Miocene tectonic boundary conditions for use in climate models. *Geochem. Geophys. Geosyst.* **9,** Q10009, doi:10.1029/2008GC002046 (2008)
50. Huber, M. & Goldner, A. Eocene monsoons. *J Asian Earth Sci*. **44,** 3-23 (2012).
51. Besse, J., Courtillot, V., Pozzi, J. P., Westphal, M. & Zhou, Y. X. Palaeomagnetic estimates of crustal shortening in the Himalayan thrusts and Zangbo suture. *Nature* **311**, 621-626 (1984).

52. Chatterjee, S., Goswami, A. & Scotese, C. R. The longest voyage: Tectonic, magmatic, and paleoclimatic evolution of the Indian plate during its northward flight from Gondwana to Asia. *Gondwana Res*. **23**, 238-267 (2013).
53. Wei, H. H., Meng, Q. R., Ding L. & Li Z.Y. Tertiary evolution of the western Tarim basin, northwest China: A tectono-sedimentary response to northward indentation of the Pamir salient. *Tectonics* **32**, 558-575 (2013).
54. Beerling, D. J. & Royer, D. L. Convergent Cenozoic CO_2 history. *Nature Geosci.* **4**, 418-420 (2011).
55. Fan, M. & Carrapa, B. Late Cretaceous-early Eocene Laramide uplift, exhumation, and basin subsidence in Wyoming: Crustal responses to flat slab subduction. *Tectonics* **33**, 509-529 (2014).
56. Fan, M., Heller, P., Allen, S. D. & Hough, B. G. Middle Cenozoic uplift and concomitant drying in the central Rocky Mountains and adjacent Great Plains. *Geology* **42**, 547-550 (2014).
57. Mix, H. T., Mulch, A., Kent-Corson, M. L. & Chamberlain, C. P. Cenozoic migration of topography in the North American Cordillera. *Geology* **39**, 87-90 (2010).
58. Sewall, J. O. & Fricke, H. C. Andean-scale highlands in the Late Cretaceous Cordillera of the North American western margin. *Earth Planet. Sci. Lett.* **362**, 88-98 (2013).
59. Carrapa, B. *et al*. Uplift of the Central Andes of NW Argentina associated with upper crustal shortening, revealed by multiproxy isotopic analyses. *Tectonics* **33**, 1039-1054 (2014).
60. Gregory-Wodzicki, K. M. Uplift history of the Central and Northern Andes: A review. *Geolog. Soc. Amer. Bull.* **112**, 1091-1105 (2000).
61. Muñoz, N. & Charrier, R. Uplift of the western border of the Altiplano on a west-vergent thrust system, Northern Chile. *J. South Amer. Earth Sci*. **9**, 171-181 (1996).
62. Gregory-Wodzicki, K. M., McIntosh, W. C. & Velasquez, K. Climatic and tectonic implications of the late Miocene Jakokkota flora, Bolivian Altiplano. *J. South Amer. Earth Sci*. **11**, 533-560 (1998).
63. Alpers, C. N. & Brimhall, G. H. Middle Miocene climatic change in the Atacama Desert, northern Chile: Evidence from supergene mineralization at La Escondida. *Geol. Soc. Amer. Bull*. **100**, 1640-1656 (1988).
64. Garzione, C. N., Molnar, P., Libarkin, J. C. & MacFadden, B. J. Rapid late Miocene rise of the Bolivian Altiplano: Evidence for removal of mantle lithosphere. *Earth Planet. Sci. Lett*. **241**, 543-556 (2006).

65. Graham, A., Gregory-Wodzicki, K. M. & Wright, K. L. Studies in Neotropical Paleobotany. XV. A Mio-Pliocene palynoflora from the Eastern Cordillera, Bolivia: implications for the uplift history of the Central Andes. *Amer. J. Botany* **88**, 1545-1557 (2001).
66. Van der Hammen, T., Werner, J. H. & van Dommelen, H. Palynological record of the upheaval of the Northern Andes: A study of the pliocene and lower quaternary of the Colombian Eastern Cordillera and the early evolution of its high-Andean biota. *Rev. Palaeobot. Palynol*. **16**, 1-122 (1973).
67. Lear, C. H., Elderfield, H. & Wilson, P. A. Cenozoic deep-sea temperatures and global ice volumes from Mg/Ca in benthic foraminiferal calcite. *Science* **287**, 269-272 (2000).
68. Zachos, J. C., Dickens, G. R. & Zeebe, R. E. An early Cenozoic perspective on greenhouse warming and carbon-cycle dynamics. *Nature* **451**, 279-283 (2008).
69. Pekar, S. F. & DeConto, R. M. High-resolution ice-volume estimates for the early Miocene: Evidence for a dynamic ice sheet in Antarctica. *Palaeogeog. Palaeoclimatol. Palaeoecol*. **231**, 101-109 (2006).
70. Cyr, A. J., Currie, B. S. & Rowley, D. B. Geochemical evaluation of Fenghuoshan group lacustrine carbonates, north-central Tibet: Implications for the paleoaltimetry of the Eocene Tibetan Plateau. *J. Geology* **113**, 517-533 (2005).
71. Wang, C. *et al*. Constraints on the early uplift history of the Tibetan Plateau. *Proc. Nat. Acad. Sci. USA* **105**, 4987-4992 (2008).
72. Xu, Q. *et al*. Paleogene high elevations in the Qiangtang Terrane, central Tibetan Plateau. *Earth Planet. Sci. Lett*. **362**, 31-42 (2013).
73. Polissar, P. J., Freeman, K. H., Rowley, D. B., McInerney, F. A. & Currie, B. S. Paleoaltimetry of the Tibetan Plateau from D/H ratios of lipid biomarkers. *Earth Planet. Sci. Lett*. **287**, 64-76 (2009).
74. Clark, M. K., Farley, K. A., Zheng, D., Wang, Z. & Duvall, A. R. Early Cenozoic faulting of the northern Tibetan Plateau margin from apatite (U–Th)/He ages. *Earth Planet. Sci. Lett.* **296**, 78-88 (2010).
75. Harris, N. The elevation history of the Tibetan Plateau and its implications for the Asian monsoon. *Palaeogeog. Palaeoclimatol. Palaeoecol.* **241**, 4-15 (2006).
76. Li, D. Temporal-spatial structure of intraplate uplift in the Qinghai-Tibet Plateau. *Acta Geologica Sinica* **84**, 105-134 (2010).
77. Song, X. Y., Spicer, R. A., Yang, J., Yao, Y. F. & Li, C. S. Pollen evidence for an Eocene to Miocene elevation of central southern Tibet predating the rise of the High Himalaya. *Palaeogeog. Palaeoclimatol. Palaeoecol*. **297**, 159-168 (2010).

78. Wang, J., Wang, Y. J., Liu, Z. C. & Li, J. Q. Cenozoic environmental evolution of the Qaidam Basin and its implications for the uplift of the Tibetan Plateau and the drying of central Asia. *Palaeogeog. Palaeoclimatol. Palaeoecol.* **152**, 37–47 1999.
79. Bershaw, J., Penny, S. M. & Garzione, C. N. Stable isotopes of modern water across the Himalaya and eastern Tibetan Plateau: Implications for estimates of paleoelevation and paleoclimate. *J. Geophys. Res*. **117**, 199-202 (2012).
80. Wang, Y. *et al*. Diet and environment of a mid-Pliocene fauna from southwestern Himalaya: Paleo-elevation implications. *Earth Planet. Sci. Lett*. **376**, 43-53 (2013).
81. Wang, C. *et al*. Outward-growth of the Tibetan Plateau during the Cenozoic: A review. *Tectonophysics* **621**, 1-43 (2014).
82. Ding, L. *et al*. The Andean-type Gangdese Mountains: Paleoelevation record from the Paleocene–Eocene Linzhou Basin. *Earth Planet. Sci. Lett.* **392**, 250-264 (2014).
83. Miao, Y. *et al*. A Late-Eocene palynological record from the Hoh Xil Basin, northern Tibetan Plateau, and its implications for stratigraphic age, paleoclimate and paleoelevation. *Gondwana Res*. **31**, 241-252 (2015).
84. DeCelles, P. G. *et al*. High and dry in central Tibet during the Late Oligocene. *Earth Planet. Sci. Lett.* **253**, 389-401 (2007).
85. Spicer, R. A., Harris, N. B. W., Widdowson, M., Herman, A. B. & Guo, S. Constant elevation of southern Tibet over the past 15 million years. *Nature* **421**, 622-624 (2003).
